# C-CTX1 and 17-OH-C-CTX1 Accumulation in Muscle and Liver of Dusky Grouper (*Epinephelus marginatus*, Lowe 1834): A Unique Experimental Study Under Low-Level Exposure

**DOI:** 10.3390/toxins18010003

**Published:** 2025-12-19

**Authors:** Yefermin Darias-Dágfeel, Andres Sanchez-Henao, Maria Rambla-Alegre, Jorge Diogène, Cintia Flores, Daniel Padilla, María José Ramos-Sosa, Paula María Poquet Blat, Freddy Silva Sergent, Salvador Jerez, Fernando Real

**Affiliations:** 1 Division of Fish Health and Pathology, University Institute of Animal Health and Food Safety (IUSA), University of Las Palmas de Gran Canaria, 35413 Arucas, Canary Islands, Spain; yeferminjesus.darias@ulpgc.es (Y.D.-D.); daniel.padilla@ulpgc.es (D.P.); maria.ramossosa@ulpgc.es (M.J.R.-S.); paula.poquet@ulpgc.es (P.M.P.B.); freddy.silva@ulpgc.es (F.S.S.); fernando.real@ulpgc.es (F.R.); 2 IRTA, Marine and Continental Waters Program, Carretera de Poble Nou Km 5.5, 43540 La Ràpita, Catalonia, Spain; maria.rambla@irta.cat (M.R.-A.); jorge.diogene@irta.cat (J.D.); 3 Mass Spectrometry Laboratory, Organic Pollutants IDAEA-CSIC, Carrer de Jordi Girona 18-26, 08034 Barcelona, Catalonia, Spain; cintia.flores@idaea.csic.es; 4 Oceanographic Centre of Canary Islands, Spanish Institute of Oceanography, Spanish National Research Council (IEO-CSIC), C. Farola del Mar 22, San Andrés, 38180 Santa Cruz de Tenerife, Canary Islands, Spain; salvador.jerez@ieo.csic.es

**Keywords:** ciguatoxins, exposure feeding, neuroblastoma 2a, cell-based assay, liquid chromatography-mass spectrometry, C-CTX1, 17-OH-C-CTX1

## Abstract

This study investigated the bioaccumulation of ciguatoxins (CTXs) in dusky grouper (*Epinephelus marginatus*) following dietary exposure to toxic fish flesh. Two feeding groups were established: group A (amberjack (*Seriola* spp.) and dusky grouper flesh) and group B (moray eel (*Muraena* spp. and *Gymnothorax* spp.) flesh). CTX-like toxicity was detected in muscle and liver of group A. Flesh toxicity progressively increased from the first sampling. In contrast, CTX activity was only detected in livers in group B. Liquid chromatography–mass spectrometry analysis revealed the presence of C-CTX1 in both groups, and the 17-OH-C-CTX1 analogue was exclusively observed in group A. Toxicity in the liver peaked at 10 weeks in experimental group A, but it showed a decline by the end of the experiment while increasing the storage of the toxin in muscle tissue. These findings demonstrate the differential bioaccumulation of CTXs in grouper flesh and liver, highlighting the potential role of the liver in metabolizing and/or detoxifying ciguatoxins. The efficacy of a combination of different techniques, including the cell-based assay (CBA) and liquid chromatography—low- and high-resolution mass spectrometry (LC-MS/MS and LC-HRMS), was demonstrated to confirm the presence of CTX analogues at very low levels. The results provide insights into CTX transfer and accumulation in marine food webs, underlining the need for further studies on toxin metabolism in predatory fish species.

## 1. Introduction

Ciguatera poisoning (CP) arises from consuming mainly fish containing ciguatoxins (CTXs), a type of toxins that are synthesized by dinoflagellates belonging to the genera *Gambierdiscus* and *Fukuyoa*. Traditionally, CTXs have been categorized based on their geographical origin [1], but a novel classification system has been developed that categorizes them based on their chemical structure [2]. To date, 19 *Gambierdiscus* species have been identified, and the Canary Islands is recognized as a hotspot, hosting at least six species [3,4].

Regarding CP, concern about its impact and health management varies depending on the location where it occurs, although this problem is widespread globally, since the European Legislation establishes that fishery products intended for consumption must not contain CTXs [5]. In addition, the European Food Safety Agency (EFSA) and the Food and Drug Administration (FDA) in the United States have established a maximum safe guidance limit for the consumption of fish contaminated with CTXs (0.01 µg CTX1B per kg) [6,7,8]. However, the Codex Alimentarius Commission has not yet established specific legal guideline values or maximum levels (MLs) for ciguatoxins in seafood [9]. The recurrence of outbreaks of CP in the Canary Islands led to its categorization as a notifiable disease in 2015 [10,11]. The mechanisms of action of CTXs are still being studied in both humans and fish, even though their symptoms are well known in humans [2]. CTXs disrupt cellular function by activating voltage-gated sodium channels (VGSCs) and causing gastrointestinal, cardiovascular, and neurological symptoms [12,13].

Furthermore, these marine toxins have different vectors capable of causing CP in humans; in the Pacific Ocean, predatory fish are the main sources responsible for CP [12]. In the Canary Islands (Atlantic Ocean), several fish species, including amberjacks (*Seriola* spp.), the dusky grouper (*Epinephelus marginatus*), the bluefish (*Pomatomus saltatrix*), and the island grouper (*Mycteroperca fusca*), have a high risk of causing CP, considering the frequency of outbreaks in which they have been involved in this archipelago. All of these species are included in a protocol established by the Government of the Canary Islands for the detection of CTXs at points of first sale, which consists of the analysis of these species, above a specified weight, before reaching the consumer [14].

Accumulation of CTXs in the tissues and subsequent magnification and biotransformation through the food web are central aspects of their transmission [2,6,15]. As CTXs ascend through the food web, they may undergo biotransformation, resulting in toxic levels for human consumption [16,17]. Different studies have explored dietary exposure to CTXs, focusing on their bioaccumulation in fish tissues and molecular alterations after ingestion [18,19,20].

Fish metabolism is crucial in CTX transformation and potential toxicity enhancement. Upon ingestion, fish can transform CTXs through oxidation to detoxify these compounds. However, certain enzymatic pathways may convert less toxic CTXs into more toxic forms, increasing the risk of intoxication at higher trophic levels, via microsomal enzymes (i.e., P450-citocrome) or cytoplasmatic enzymes (i.e., ketoreductases) [21,22,23]. This metabolic interaction explains why larger fish, such as groupers and barracudas, present greater health risks for humans, even after extended periods without new CTX exposure. This fact highlights biomagnification of CTXs and metabolites within the food chain. The accumulation of more toxic CTX forms complicates the prediction of safe fish for consumption, challenging management efforts [24].

Dusky grouper (*E. marginatus*) is a significant fish species in the Canary Islands due to its importance in commercial and recreational fishing. Found throughout the northeastern Atlantic, this carnivorous species, part of the Serranidae family, preys on crustaceans, molluscs, and fish, including other predatory fish. This species is also known for its sedentary nature, often staying in one area for extended periods [25,26]. As a main predator, the dusky grouper plays a crucial role in regulating the balance of ecosystems, particularly in rocky or rugged seabeds [27]. Despite the socio-economic importance of this species, and the fact that its reproductive cycle has been successfully closed, it is not cultivated on a regular basis [28]. So, given its position at the top of the food web in the Canary Islands and its frequent association to the CP, the dusky grouper is relevant to understanding how CTX bioaccumulation occurs in marine ecosystems. The risk of CP is managed through the Official Control Protocol of Ciguatoxins in fish for consumption maintained in this region, which requires testing of all groupers over 8 kg before sale [14]. From 2008 to 2022, the Canary Islands Epidemiological Surveillance Service recorded CP outbreaks, with four events directly associated with *E. marginatus*, regarding sport or illegal fishing [10]. A fifth outbreak linked to this species occurred in 2024 [29]). The University Institute of Animal Health and Food Safety at the University of Las Palmas de Gran Canaria (ULPGC) analyzed the last three specimens that caused CP outbreaks, where they were found to have a range of toxicity from 0.255 to 0.333 ng Eq. of CTX1B/g of flesh [29].

Groupers frequently feed on moray eels in the wild, and there is a close relationship between the amount of CTXs that the two species can accumulate in the natural environment [30]. This is the reason why further research needs to be conducted to determine the CTX transmission capacity between both species and what tissues are most involved in the accumulation process.

Considering all of the above, it is necessary to investigate the effects of CTX bioaccumulation, using the toxicity levels found in nature, in dusky grouper, a species that is highly involved in the CTX transmission.

The primary objective of this study is to assess the transfer of CTXs through the controlled exposure of adult *E. marginatus*—a species and age class rarely available for experimental research—to toxin concentrations comparable to those reported in fish from the natural marine environment of the Canary Islands. Given the low toxin concentrations used, the study also aims to evaluate the utility of combining different analytical techniques, including the cell-based assay (CBA) and liquid chromatography–mass spectrometry (LC-MS), using both low- and high-resolution mass spectrometry (LC-MS/MS and LC-HRMS) to characterize the toxin profile and determine the toxic potential of the samples. This unique approach will be useful to investigate the presence and the spatial distribution of CTXs in liver and muscle tissues throughout the experimental period.

## 2. Results

The food in both feeding groups was classified as toxic by the CBA assessment, with about a 7-fold difference in toxicity between food supplied to group A and group B (Table 1).

### 2.1. CTX Accumulation in Muscle and Liver Tissues (by CBA)

CTX-like toxicity was revealed in both flesh and liver samples from experimental groups at some point in the trial. All measures are summarized in Table 1.

In all sampling weeks of group A, toxicity was observed in the muscle tissue of groupers, except in sampling week 4, where toxicity was below the limit of detection (LOD) in one of the fish (Table 1). The values of toxicity detected in the muscle in group A tend to increase progressively and linearly over the study period.

**Figure 1:**
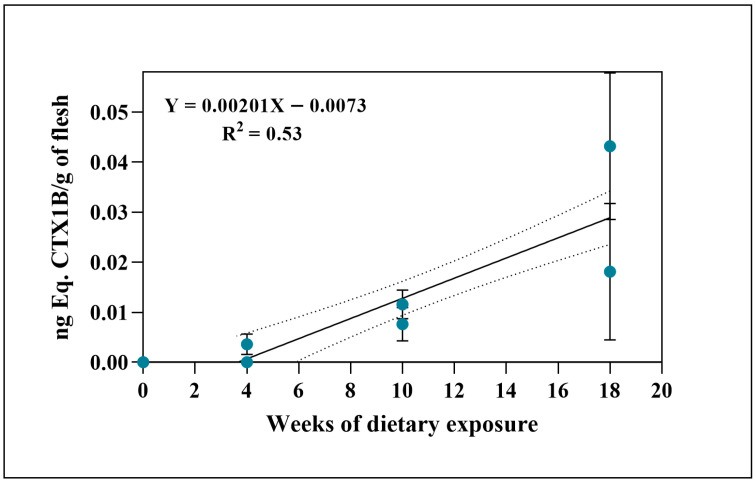
CTX-like toxicity (ng Eq. CTX1B/g of flesh) values in flesh per fish by sampling week (4, 10, and 18) from group A, assessed by CBA. The dashed lines indicate the 95% confidence interval of the linear fit.

Thus, in the second sampling, corresponding to 10 weeks of continuous feeding (6 days per week) (Figure 1), higher quantification levels in flesh were measured compared to the first 4 weeks (approximately 2.6 times higher, regarding the average toxicity levels recorded at each sampling point). Then, the highest levels of toxicity in this tissue were obtained in week 18, approximately 3.2 times higher than sampling week 10 regarding toxin accumulation. On the other hand, no CTX-like toxicity was detected in the flesh of the experimental individuals of group B (Table 1).

Regarding the analysis in livers, toxicity was detected in all the specimens in the study in both experimental groups (A and B) (Table 1).

**Figure 2:**
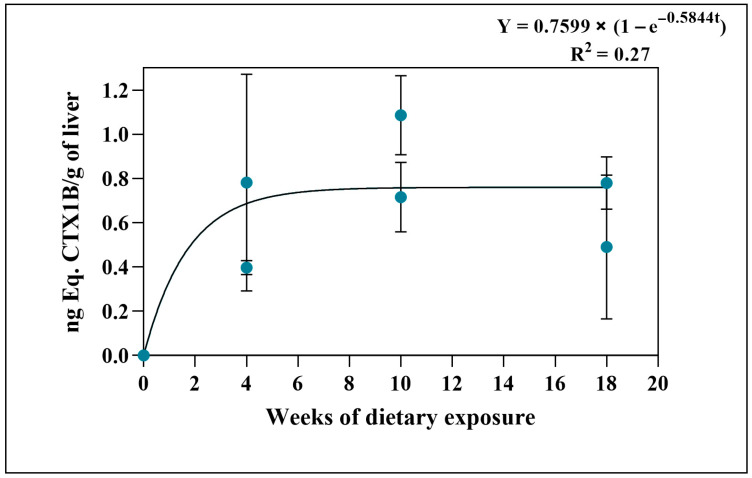
CTX-like toxicity (ng Eq. CTX1B/g of liver) values in liver per fish by sampling week (4, 10, and 18) from group A, assessed by CBA.

For group A, toxicity levels were detected from the first sampling at 4 weeks of feeding. In the second sampling (week 10), the peak of toxicity was reached in the livers (approximately three times more toxic), and in this group, the fish livers from week 18 of feeding were slightly less toxic than those from week 10 of feeding, evidencing an apparent saturation of the trial (Figure 2). The livers of experimental group B showed a moderate toxicity ranging from an average of 0.044 ng Eq. CTX1B/g of liver in the first sampling in week 6 to more than double in week 12 (Table 1, Figure S1). Additionally, no detectable amounts of toxins were obtained in the flesh or liver of the initial or final controls.

No statistically significant differences were observed between CTX concentrations within sampling days for muscle and/or liver samples (*p* > 0.05).

Other parameters related to toxin ingestion and storage in the different tissues were calculated. On the one hand, the total toxin intake was calculated as the total food intake offered and accepted throughout the experiment in relation to the CTX content in the feed. On the other hand, the total toxin burden in each tissue was estimated using the CTX-like toxicity and the full weight of the respective tissue. The relation between the previous parameters allowed us to estimate the percentage of the ingested toxins that are accumulated in each tissue (apparent assimilation efficiency), assuming that there are no changes in the composition of these toxins (Table 1).

The ciguatoxin concentration obtained in the tissues allowed for the toxicokinetic analysis of assimilation (compiled in Table S1). The liver toxicities of group A best fit an exponential model with a plateau (Y = A × (1 − e^−kt^)), suggesting fast initial uptake followed by a tendency towards saturation (t_90_ ≈ 3.9 weeks). In contrast, the toxicity levels achieved in the muscle of group A did not suggest that the tissue had reached its maximum capacity to accumulate CTXs, so the best fit applied was a linear model (uptake rate = 2.0 pg/g/week). For group B, the sample size only allowed a linear fit (uptake rate = 27.1 pg/g/week) (Table S1).

**Figure 3:**
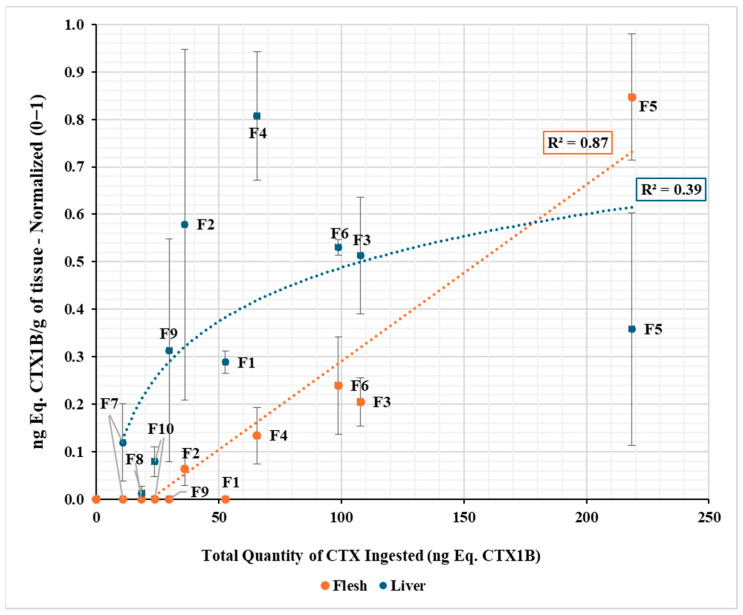
Relationship between the total amount of toxin ingested (ng CTX1B Eq.) and the concentration of the toxin in the liver (blue) and muscle (orange) assessed by CBA. Data correspond to both experimental groups (A and B) and are represented on a unified axis of cumulative dose, independent of exposure time. Each point represents the individual fish (F no.). The best fits obtained were for the liver logarithmic model, R^2^ = 0.39, and for the muscle linear model, R^2^ = 0.87.

The relationship between the total amount of toxin ingested and CTX concentrations in the liver and muscle was analyzed by integrating the data from both experimental groups (A and B) so that the variables were represented as a function of the cumulative dose rather than the exposure time (Figure 3). When normalizing the values for both tissues, it was observed that the liver CTX concentrations showed a pronounced increase at ingested amounts, and this tendency was not maintained at higher amounts. The best fit corresponded to a logarithmic model (R^2^ = 0.39), with a high dispersion between individuals. By contrast, in muscle, the CTX concentration increased progressively and proportionally to the amount ingested (R^2^ = 0.87), with lower dispersion and high correlation between the total ingested amount of toxin and accumulated concentration. Furthermore, for these data, a significantly positive correlation was seen between the concentration of toxin in both liver and muscle tissues and the amount of toxin ingested (*p* < 0.001, for both tissues) (Figure 3). Moreover, the data showed a positive correlation between feeding time and flesh toxin burden (*p* = 0.003).

### 2.2. LC-MS/MS and LC-HRMS Analysis

**Figure 4:**
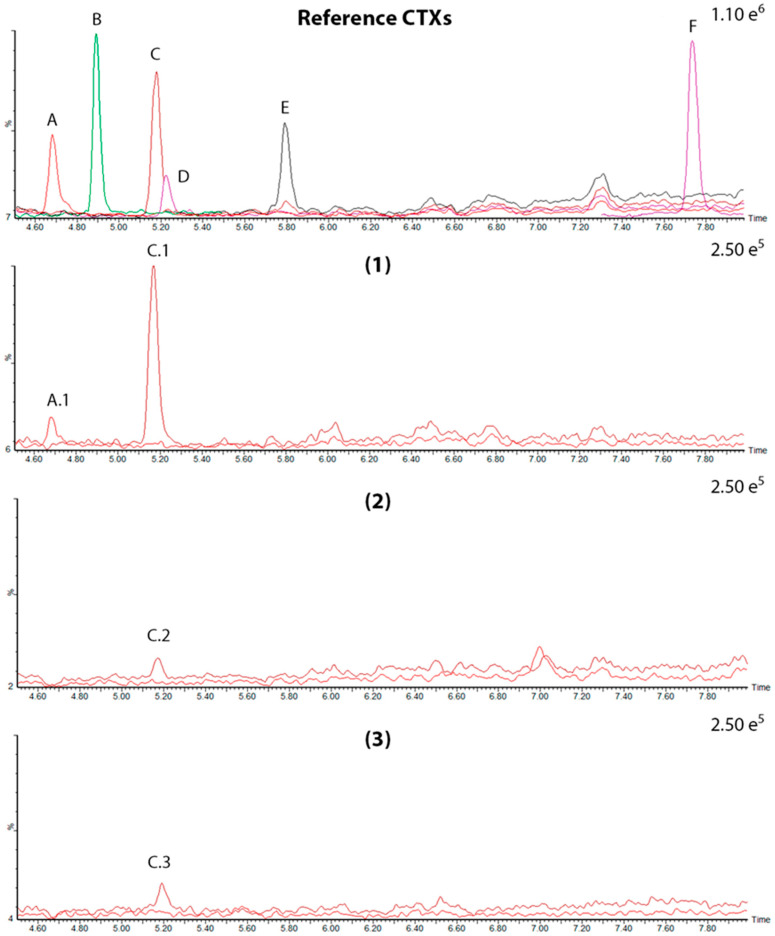
Extracted ion chromatograms (XIC) of the CTXs studied. (Reference CTXs) Standard solutions and internal reference material (IRM) (A) 17-OH-C-CTX1 in a fish liver (*Seriola* sp. IRM); (B) CTX1B standard solution; (C) C-CTX1 in a fish liver (*Seriola* sp. IRM); (D) 17-OH-56-CH_2_-C-CTX1 in a fish liver (*Seriola* sp. IRM); (E) 56-CH_2_-C-CTX1 in a fish liver (*Seriola* sp. IRM); and (F) CTX3C standard solution. (1) CTXs detected in experimental fish liver no. 3 from group A: (A.1) 17-OH-C-CTX1 and (C.1) C-CTX1. (2) CTXs detected in flesh from experimental fish no. 5 from group A: (C.2) C-CTX1. (3) CTXs detected in liver from experimental fish no. 9 from group B: (C.3) C-CTX1.

Mass spectrometry assessment in basic mobile phases confirmed the presence of C-CTX1 in the food provided to experimental group A, in a concentration of 0.0132 ± 0.0001 ng Eq. CTX1B/g of feed. Moreover, in a different study performed with this pool sample, traces of 17-OH-C-CTX1 were also detected [31]. The low concentration of CTXs in the group B food sample prevented its determination by this methodology (<LOD). Additionally, the LC-MS/MS assessment in the sampled extracts sent to the Institute of Agrifood Research and Technology (IRTA) facilities demonstrated the presence of at least two analogues of the C-CTXs group, the C-CTX1 and the 17-OH-C-CTX1, in experimental grouper tissues (Figure 4, Figures S2 and S3). C-CTX1 was the main analogue in the samples where CTXs were detected; on the contrary, the 17-OH-C-CTX1 analogue was only evidenced in the most toxic tissue samples (Table 2).

Regarding group A, C-CTX1 was only detected in flesh from one of the fish fed for 18 weeks. By comparison, in all liver samples from this experimental group, C-CTX1 was detected at different levels (from 0.025 to 0.167 ng Eq. CTX1B/g of liver). In relation to the 17-OH-C-CTX1 analogue, this toxin was only evidenced in liver samples from this experimental group (Table 2).

In group B, LC-MS/MS analysis demonstrated C-CTX1 presence in liver tissues. Instead, all toxic flesh were <LOD. Furthermore, no traces of 17-OH-C-CTX1 were detected in the tissues from this experimental group.

**Figure 5:**
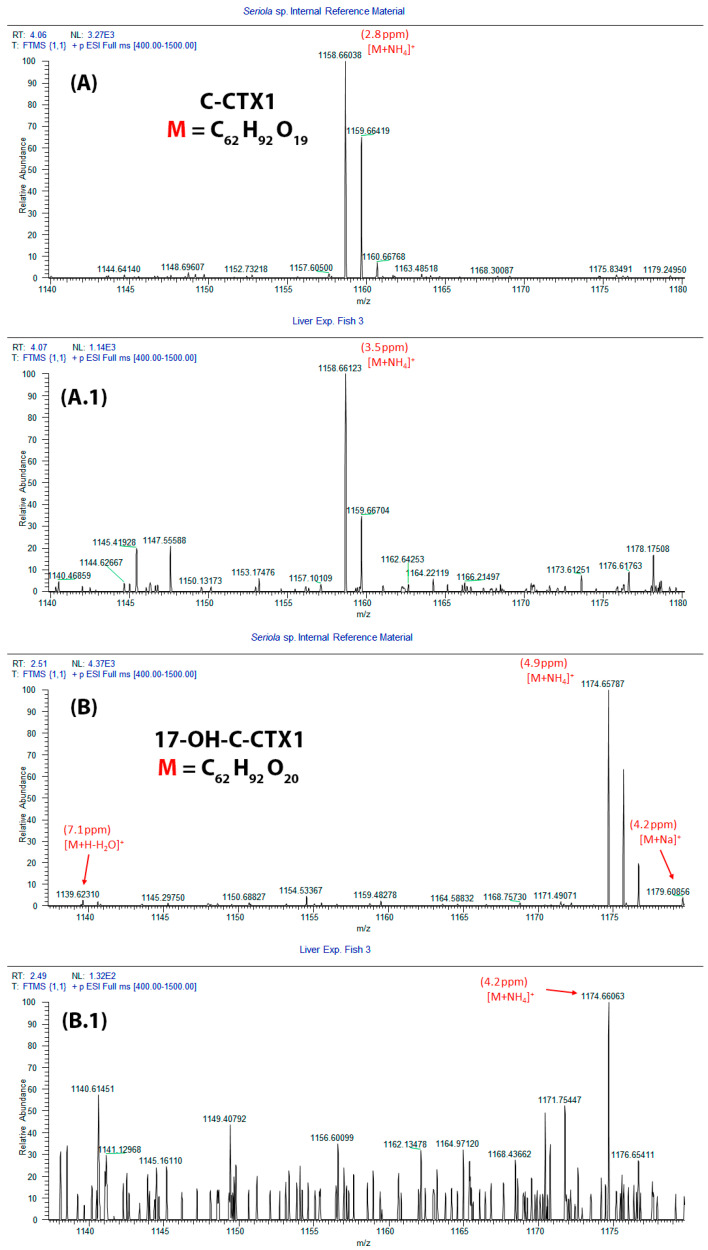
HRMS spectra (isotopic pattern) of (**A**) C-CTX1 [M + NH_4_]^+^ in flesh from *Seriola* sp. IRM; (**A.1**) C-CTX1 [M + NH_4_]^+^ in liver from experimental grouper no. 3; (**B**) 17-OH-C-CTX1 ([M + H]^+^ + [M + NH_4_]^+^ + [M + Na]^+^) in flesh from *Seriola* sp. internal reference material; (**B.1**) extracted ion chromatogram of 17-OH-C-CTX1 [M + NH_4_]^+^ in liver from experimental group no. 3.

The LC-HRMS assessment detected CTX signals in three liver samples from experimental group A. These signals matched the retention time of C-CTX1 and 17-OH-C-CTX1 of the CTXs observed in *Seriola* sp., which was used as reference (Figure 5) (further details are included in Table S2), confirming the signals detected by LC-MS/MS.

**Figure 6:**
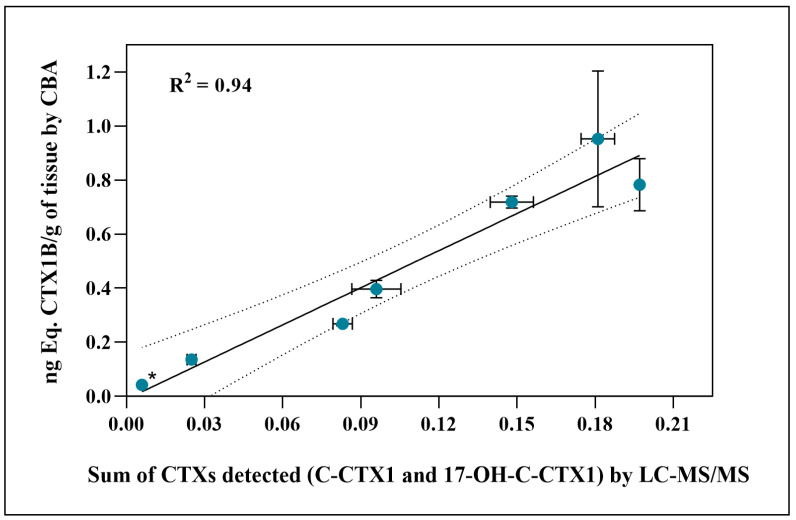
Correlation between liver and flesh toxicity detected by CBA and the sum of analogues detected by LC-MS/MS of group A. Linear regression with coefficient of determination of R^2^ = 0.94. (*) C-CTX1 was detected in the flesh only in fish no. 5 (toxicity and LC-MS/MS data belonging to the first replicate of extracts). The dashed lines indicate the 95% confidence interval of the linear fit.

In a further analysis, in the first replicate of extracts from experimental group A, a significant correlation was found between the sum of the two analogues identified by LC-MS/MS and their results of CTX-like toxicity by CBA values (*p* < 0.001) (Figure 6).

## 3. Discussion

The selection of fish specimens for this study was based on several factors. Dusky grouper (*E. marginatus*) holds significant fishing value in the Canary Islands [32] and plays a key role in CTX bioaccumulation within the marine food web, as supported by numerous CP cases reported in the region [2,10]. The availability of adult specimens of this species—seldom accessible for experimental research—further provided a rare opportunity.

CTXs are lipophilic polyether compounds that cause poisoning at low concentrations present in fish, and they can persist in their tissues for months or years in naturally exposed individuals [33]. In this regard, the FDA and EFSA have set safety thresholds of 0.01 ng/g for CTX1B and 0.1 ng/g for C-CTX1, which were calculated by the intraperitoneal injections of these toxins into mice [6]. However, these limits are considered insufficient and may change as more data on new CP outbreaks become available [2,34,35].

Because of these challenges, experimental studies often require fish that have not been previously exposed to CTXs, typically relying on freshwater species or marine species raised from postlarvae or juveniles [36,37,38]. In the present work, the used of adult groupers born and bred in captivity [39]—therefore having no previous contact with CTXs—allowed us to address the constraints while avoiding the ethical implications of removing an endangered species from the wild. Moreover, the sample size employed was sufficient to identify accumulation trends, as suggested by recent research and new trends in animal experimentation [40]. The fish used in this study were all females in an early stage of maturation. It should be noted that this species is a protogynous hermaphrodite. Although it was not the objective, it was not possible to evaluate the bioaccumulation of CTXs according to sex, as other studies have proven [41].

This study developed an experimental model simulating natural exposure of *E. marginatus* to CTXs under controlled conditions, using food prepared from wild toxic fish captured in the Canary Islands. The toxin levels applied in the experimental diet reflected those present in the natural food web in which the dusky grouper belongs.

In group A, flesh toxicity levels increased progressively in each feeding period, whereas in group B, toxin levels remained below the detection limit (Table 1). The positive trend between the time of food exposure and toxin concentration in muscle in experimental group A (Figure 1) indicates a gradual accumulation of CTXs. The observed pattern suggests an initial transfer of dietary toxins to the liver, followed by slower storage in muscle tissue (Figure 1 and Figure 2). Previous studies have shown that the ingestion of ciguatoxic food leads to intestinal absorption, entry into the blood system, and subsequent hepatic distribution [20], where the highest concentrations are typically found [42,43]. Our results are consistent with these findings, while also showing that progressive increases in muscle detected by CBA agree with results from other experimental observations [37,42,44,45,46]. As grouper is a slow-growing species [47], toxin concentrations in muscle are unlikely to be influenced by somatic growth dilution in contrast to the results obtained with juveniles in their exponential growth phases [44]. In experimental group A, dusky groupers sampled at week 4 displayed high toxicity levels in their liver, more than 200 times higher than muscle, reaching the maximum average concentration in this tissue at week 10. By the final sampling, however, liver toxicity did not continue to rise as sharply, reducing the liver-to-muscle difference to about 20-fold (Figure 2 and Table 1). This pattern aligns with previous reports identifying the liver as the main organ with the highest concentration of CTXs in *E. marginatus* specimens from the Canary Islands waters [43] but highlights a clear difference in accumulation trends between flesh and liver with the experimental conditions (Figure 3). This divergence is consistent with the liver’s role as the primary site of CTX biotransformation. The higher variability observed in hepatic concentrations likely reflects inter-individual differences in detoxification dynamics rather than analytical error, suggesting that, beyond a certain exposure threshold, liver toxin burden may fluctuate due to enhanced metabolic process or redistribution to peripheral tissues. A recent study has shown that fish hepatic microsomes can conjugate C-CTX-1/-2 via UGT-mediated glucuronidation, a phase II pathway not detected in mammalian microsomes. Such conjugation may facilitate detoxification or elimination of CTXs, contributing to lower persistence in muscle compared to liver. Overall, these findings support the notion that hepatic metabolism likely influences organ-specific CTX distribution in the dusky grouper [48].

In groupers from experimental group B, toxicity was evidenced in their liver tissue from the first sampling at week 6, with higher values than those present in the food ingested and increasing substantially until the final sampling at week 12. This pattern resembles that of experimental group A, but on a lower toxicity scale. In contrast, the total amount of toxin ingested by this group was insufficient to promote its distribution to muscle, remaining below CBA detection limits (Table 1). Similar findings have been reported in other fish species, such as lionfish (*Pterois volitans*), where CTXs were detected in liver but not in muscle [38]. Nevertheless, our study differs in design as it involves long-term dietary exposure at relatively low toxin doses. To the best of our knowledge, this is the longest dietary exposure to CTXs assessed to date in an adult predatory fish species under such low doses of CTX intake. Other experimental models have generally been shorter in duration, including 20 days of the C-CTX1 accumulation phase and 14 weeks of non-toxic feeding [46], 16 weeks in juvenile herbivorous [44], 11 weeks in freshwater fish [37], or 8 weeks feeding an orange-spotted grouper (*E. coioides*) [42].

In the frame of the experimental conditions conducted, the results are consistent with the theory that ciguateric fish in the wild do not show clear signs of poisoning [49]. However, it is noteworthy that the toxicities reached in the livers were about 1 ng Eq. CTX1B/g, and this same tissue and fish species (*E. marginatus*) has reached levels of 4.9 ng Eq. CTX1B/g in the waters of the Canary Islands [43]. Considering this fact, we hypothesize that different amounts of CTXs in the food, the frequency of feeding on ciguatoxic preys in the wild, and metabolization of the toxins in periods of CTX-free feeding could influence these differences.

In relation to tissue assimilation, our experimental design and results are comparable to those reported for *Lagodon rhomboides*, an omnivorous Caribbean fish species exposed to C-CTX1-contaminated food and analyzed using the same CBA approach [46]. Therefore, the calculated assimilation rates (by linear regression) and toxin intake doses can be normalized to allow comparison of the assimilation rate per unit of C-CTX ingested in liver and muscle tissues for both fish species (after unit adjustment, see Table 3).

This comparison revelated that muscle tissue in *L. rhomboides* exhibited ~1.5 times assimilation efficiency of *E. marginatus*, whereas *E. marginatus* displayed a 5- to 6-fold higher hepatic assimilation capacity. This marked difference in liver accumulation warrants further investigation to elucidate the metabolic mechanisms underlying this enhanced efficiency, which may be linked to species-specific trophic level or physiological traits.

Similar findings were reported for *E. coioides* [42], where liver consistently showed higher concentrations and incorporation rates. However, in that study, fish were exposed to different P-CTX congeners, which displayed distinct uptake patterns. Therefore, when comparing experimental models, differences between CTX families and even among analogues withing the same family must be carefully considered.

The experimentally determined accumulation efficiencies obtained here (Table 1) align closely with recent quantitative models of CTXs bioaccumulation [50,51]. These models suggest that although CTXs can bioaccumulate in fish, they do not necessarily biomagnify systematically across trophic levels. Key processes cited include somatic growth dilution, depuration, dietary variability, and tissue-specific toxicokinetics, which together can markedly limit the eventual burden in edible muscle, even under continuous exposure. Importantly, when compared with data from another experimental species (see Table 3), a pronounced fish-tissue-specific partitioning is underscored: even with high hepatic uptake, muscle accumulation remains limited. Thus, our findings reinforce the view that muscle CTX concentrations, even when exposure is sustained, may remain relatively low, while other tissues (e.g., liver) act as temporary reservoirs. This differential tissue distribution helps explain why wild fish could show low or undetectable CTX levels in flesh despite ongoing environmental exposure. The LC-MS/MS assessment confirmed the presence of C-CTX1 in feed provided to experimental group A. In addition, in a previous work, the presence of traces of 17-OH-C-CTX1 was found in this pool [31]. This latest analogue was reported in amberjacks linked to CP incidents int the Canary Islands, being the second most abundant analogue presented in samples from this geographical area [52]. In liver extracts from experimental group A, C-CTX1 and 17-OH-C-CTX1 were identified, providing valuable information about the pharmacokinetic of CTXs in the dusky grouper. By contrast, no LC-MS signals of monitored CTXs were detected (<LOD) in the food provided to experimental group B, although CTX-like toxicity was confirmed by CBA. The detection of C-CTX1 in both groups reinforces its role as a key component in CTX bioaccumulation in predatory fish from this geographical area [52,53]. The stable ratio of 17-OH-C-CTX1 to C-CTX1 (17% of the quantification obtained for C-CTX1) in livers from group A explains its absence in some individual extracts (e.g., fish extracts no. 5 or liver extracts from group B) as values would have fallen below the LOD (0.008 ng Eq. CTX1B/g) (Table 2). The differences in analogue ratios compared with naturally contaminated grouper samples [52] may be attributable to tissue type (e.g., liver vs. muscle) or variability at low concentrations.

In summary, the liver emerged as the primary organ involved in the fate of absorbed CTXs (Figure 3), consistent with the literature that describes not only accumulation but also clearance or conversion into less or more toxic forms [22,54]. However, no evidence of CTX biotransformation processes was found in this study since the same two analogues detected in the feed supplied to the fish in experimental group A were later identified in their tissues. This raised the possibility that biotransformation may occur mainly across trophic levels (from lower to higher), rather within top predators, as occurred in this experimental model. Further studies are required to confirm the total absence of biotransformation, as supplied CTX could be transformed into other unknown analogues.

Furthermore, the significant correlation observed between the CBA quantification and LC-MS/MS detection suggests that the toxic potential revelated by cytotoxicity assay corresponds to the analogues identified (C-CTX1 and 17-OH-C-CTX1) (Figure 6), which is in agreement with previous reports [55].

Finally, although LC-HRMS generally offers higher mass accuracy, it was of limited use for low-toxicity samples in CTX research due to its relatively high detection limits. Nonthesis, the clean-up strategies and concentrations applied allowed us to detect signals compatible with C-CTXs. However, these signals alone did not meet the strict criteria required for confirmation. In contrast, the LC-MS/MS methodology, characterized by lower detection limits, enabled the confirmation of these signals when used in combination with LC-HRMS. This complementary approach overcame the individual limitations of each technique.

Altogether, the integration of CBA, LC-MS/MS, and LC-HRMS in this study yielded satisfactory results in assessing the overall CTX-like toxicity of contaminated samples with relatively low toxicity levels, identifying the CTX analogues present.

## 4. Conclusions

This study highlights CTX bioaccumulation in the liver and muscle of dusky grouper (*E. marginatus*), with the liver playing an important role in the temporal storage of the toxins, subsequently leading to its distribution to muscle. To the best of our knowledge, this is the first study to quantify CTX uptake values in full-growth predatory fish species subjected to dietary exposure periods as long as 12 and 18 weeks under intake levels comparable to those found in the wild.

This work also confirmed the power of chromatographic techniques, particularly LC-MS/MS, in detecting C-CTX analogues at low concentrations, and underscores the importance of bioaccumulation processes, which play an important role in the overall toxicity of predatory fish. Moreover, the combined use of CBA, LC-MS/MS, and LC-HRMS has proven to be highly effective in evaluating the toxicity of a real sample while accurately identifying the CTX analogues it contains. This integrated approach offers a reliable toolkit for the characterization of CP, contributing to improved food safety and public health protection.

## 5. Materials and Methods

### 5.1. Fish, Experimental Design, and Maintenance Conditions

The specimens used were adult groupers (n = 16) born and raised in tanks at the Centro Oceanográfico de Canarias-Instituto Español de Oceanografía (IEO-CSIC), where they were maintained for over seven years under controlled conditions, thus ensuring no prior exposure to CTXs. The average weight and length were 1480.3 ± 463.9 g and 41.1 ± 4.6 cm, respectively. Groupers were placed in 1 m^3^ tanks individually under natural conditions of temperature (21.8 ± 1.5 °C), salinity, and photoperiod, ensuring constant water exchange and adequate aeration. Prior to the experiment, a conditioning period was carried out to the new daily experimental diet since previously, the fish were fed frozen mackerel 3 times a week and another group was fed commercial feed.

The experimental model was designed with two groups of fish, experimental (n = 10) and control (n = 4), and two fish were established as initial controls (before the conditioning period) (n = 2). Each group, after the conditioning period, was divided into two subgroups based on the diet they would receive. One subgroup was fed a mix of toxic amberjack and dusky grouper flesh, named group A (n = 6), and the other was fed toxic moray eel flesh, identified as group B (n = 4). The control group was also split into 2 subgroups, including one fed amberjack flesh (control group A) and the other fed moray eel flesh (control group B); the flesh was previously determined without CTX-like activity (n = 2 for each control group) (Figure S4 at Supplementary Materials).

Fish were fed toxic or non-toxic flesh for 18 weeks in group A and 12 weeks in group B. Sampling was scheduled according to the weeks of dietary exposure time, with two replicates per point (n = 2). Group A was sampled at weeks 4, 10, and 18, and group B was sampled at weeks 6 and 12. Control groups were sampled on day 0 and at weeks 18 (group A) and 12 (group B), respectively (Figure S4). The trial design can be found in more detail in a previous work with other results of interest [56].

This experimental design was previously approved by the Ethics Committee for Ani-mal Welfare of IEO-CSIC (1236/2022) and, finally, authorized by the Department of Agriculture, Livestock, Fisheries and Water of the Canary Islands Government. The number of animals used was not very large, and it was chosen following the 3R principle and the current trend in minimization of experimental animals to observe differences [40]. This number is enough to observe different trends between groups. The specimens used, adult *E. marginatus,* belong to a fish species linked to cases of CP. Moreover, the fact that they were born and raised in captivity allow us to be fairly certain that they had never been exposed to CTXs [39], and as adults, they do not present the susceptibility of larvae or juveniles to toxic exposure. Thus, despite the limited number of fish available, they constituted a group of incalculable value for our objective. Importantly, access to such specimens is highly unusual, representing a rare experimental opportunity that is unlikely to be easily replicated in the near future.

### 5.2. Food Preparation and Ciguatoxin Dietary Exposure

Experimental feeding of groupers involved homogenizing different flesh portions of toxic flesh from 12 amberjacks and 6 dusky groupers (for group A) and 8 moray eels (for group B). All of these toxic fish used as food were caught in the Canary Islands waters. Fish samples used to prepare the group A feed were characterized by CBA and LC-MS/MS within the EUROCIGUA project (GP/EFSA/ASCO/2015/03). For group B, different species of moray eel (*Muraena augusti* and *Gymnothorax unicolor*) were purchased from local fishmongers. After removing the skin and connective tissue, the raw flesh was mixed and homogenized. The toxic potential of flesh homogenates was assessed by CBA, resulting in CTX-like toxicities of 0.109 ± 0.003 ng Eq. CTX1B/g of flesh for group A and 0.015 ± 0.001 ng Eq. CTX1B/g of flesh for group B.

A prior conditioning period was necessary for the new food, and during the trial, groupers were fed 6 days a week at a ratio of 1.3% of their biomass. Corresponding to this daily ration ingested, and according to the determination of CTXs obtained in the flesh, group A groupers received 0.0014 ng Eq. CTX1B/g of fish weight, and group B groupers received 0.0002 ng Eq. CTX1B/g of fish weight in their daily food (the quantity of daily CTX ingestion was determined in relation to the CTX concentration present in the feed and the amount of feed ingested). Therefore, the CTX concentrations used in the feed correspond to toxin levels commonly found in the marine environment, as determined by the Official Control Protocol of Ciguatoxin applied by the Canary Islands Government [14]. Each ration was administered individually as the fish were maintained in single separate tanks.

### 5.3. Reagents and Standard Solutions

All solvents employed during sample extraction and Solid Phase Extraction (SPE) procedures and mixtures were of high-performance liquid chromatography (HPLC) grade, except the final methanol step from each procedure, which was of LC-MS grade; additionally, ultrapure water (resistivity > 18 MΩ cm) was obtained from a Milli-Q^®^ water purification system (Millipore Ltd., Billerica, MA, USA). Chromatographic assessment was carried out with LC-MS-grade chemical reagents.

CTX1B and CTX3C standard solutions for the assessment of recovery and matrix effect on SPE, and calibration purposes by LC-MS, were provided by Institute Louis Malardé (ILM, French Polynesia). As no standard solution for C-CTXs are commercially available, an extract containing C-CTX1, 56-methoxy-C-CTX1, 17-hydroxy-C-CTX1, and 17-hydroxy-56-methoxy-C-CTX1 analogues, from an amberjack (*Seriola* sp.) involved in a CP outbreak that occurred in the Canary Islands in 2023 [52], was used for retention time and ion ratio confirmation. Additionally, an extract containing C-CTX1/2 and C-CTX3/4 was received as a gift from Alison Robertson (University of South Alabama, USA), IRTA (Tarragona, Spain) facilities, and was used for the development of the LC-MS/MS method and retention time confirmation of these toxins.

### 5.4. Extraction of CTXs

The extraction protocol was conducted as described by Yogi et al. [57] and modified by Tudó et al. [58], applying adaptations according to the needs of our laboratory. The protocol consisted of processing 10 g of fish tissue that was cooked at 70 °C for 10 min. The CTXs were extracted twice in 20 mL of acetone, and the flesh was ground in Ultra-Turrax at 17,500 rpm. The supernatant was recovered after centrifuging at 3000× *g* for 10 min at 4 °C; then, 40 mL of pooled supernatant was filtered with a 0.45 µm PTFE filter and evaporated in a rotary evaporator at 55 °C to a dry residue. After this extraction, a liquid/liquid partition was carried out with organic solvents, twice with diethyl ether mixed with water (1:4); these two organic fractions were evaporated on rotary evaporation. After that, a deffating phase separation was carried on with methanol/water (80%) and *n*-hexane (1:2). A second *n*-hexane wash was performed, discarding the first part and adding *n*-hexane again. The methanolic phase was dried out under N_2_ current at 40 °C. Finally, the dry residue was resuspended in 4 mL of methanol and stored at −20 °C until further assessment by CBA and clean-up procedures for LC-MS analysis. Four extracts of flesh and two of liver were prepared from each fish.

### 5.5. Clean-Up Procedures

For LC-MS assessments, all extracts had to be cleaned up by SPE in order to reduce the coextractives causing matrix effects. For this purpose, one sample extract of each tissue per fish from group A and their corresponding feed extract were cleaned up with the SPE, as described by Spielmeyer et al. [59], with slight modifications. Briefly, 3.5 mL of methanolic sample extract was dried out and reconstituted in methanol/water (80%) (the 0.5 mL remaining from the initial extract was reserved for cell-based assay) and passed through the reverse-phase SPE Polar-modified polystyrene-divinylbenzene (PDVB) copolymer cartridge (Macherey-Nagel^®^ CHROMABOND^TM^ EASY, Düren, Nordrhein-Westfalen, Germany, 200 mg/3 mL) previously conditioned with acidified ethyl acetate (3 mL), acetonitrile (6 mL), and methanol/water (80%) (9 mL); the sample retained in the SPE cartridge was eluted using acetonitrile (3 mL) and dried out under N_2_ current. The dried residue was resuspended in acidified n-hexane–ethyl acetate (1:1) (2 mL) and passed through a normal-phase Silica cartridge (Agilent^®^ HF Bond Elut-SI, Santa Clara, CA, US, 500 mg/3 mL) previously conditioned with acidified methanol–ethyl acetate (1:3) (3 mL), acidified ethyl acetate (6 mL), and acidified n-hexane–ethyl acetate (1:1) (9 mL); the filtered fraction was recovered for LC-MS evaluation, and the sample retained in the cartridge was eluted with acidified ethyl acetate and acidified methanol–ethyl acetate (1:3) (7 mL). Both the filtrate and the eluate were then dried out under N_2_ current and resuspended in 500 µL of methanol (LC-MS grade), giving a final concentration of 17.5 g Eq. of tissue/mL. The correction factor for the recovery of this cleaning up, applied for the calculation of the concentration of C-CTXs analogues in the different tissues, was 78% [31].

Regarding group B, the SPEs were conducted according to Murata and Yasumoto [60], with modifications proposed for the first time in this study. Detailly, 3.5 mL of one methanolic sample extract per tissue from each fish and their feed sample extract were transferred to a glass tube and dried out under N_2_ current (the remaining 0.5 mL was reserved for the cell-based assay). Then, the sample was reconstituted firstly in 500 µL of acetone and agitated by vortex. After that, 2 mL of *n*-hexane was added and agitated again for a final proportion of *n*-hexane–acetone (4:1). Afterwards, the glass tube was placed in a sonication bath for 5 min. The sample was passed through a normal-phase SPE Florisil column (J.T.Baker^®^, Radnor, PA, US, 500 mg/3 mL) at a flow of 0.6 mL/min, which was previously conditioned with 3 mL of *n*-hexane–acetone (4:1). Once the sample was placed in the Florisil cartridge, another 600 µL of acetone was added in the glass tube and agitated by vortex. Next, 2.4 mL of *n*-hexane was added to the glass tube after mixing with vortex, and the glass tube was then placed in a sonication bath for 3 min. This new mix of *n*-hexane–acetone (4:1) was finally passed through the Florisil column. The filtered fraction of *n*-hexane–acetone (4:1) was recovered and considered for LC-MS analysis. Then, CTXs retained in the cartridge were eluted using 3 mL of acetone–methanol (9:1) at a flow of 1 mL/min (the sample was eluted until the column was completely dry). Both fractions were dried out and reconstituted in 500 µL of methanol (LC-MS grade) to reach a final concentration of 17.5 g Eq. of tissue/mL. Thanks to the modifications in this SPE protocol, the recovery obtained raised from 67% and 103% [31] to 95.2% and 100% for CTX1B and CTX3C, respectively. Subsequently, the enhanced recovery data of CTX1B was employed in the analysis of C-CTX analogues.

In both groups, grouper flesh and feed cleaned-up extracts were subsequently concentrated at 70 g tissue Eq. of sample/mL for LC-MS analysis.

### 5.6. Neuro-2a Cell-Based Assay for CTX Determination

The toxicity of the feed and fish tissue samples of the groupers was assessed by the Neuro-2a cell-based assay. For the assay and cell maintenance, we followed the protocol described by Caillaud et al. [61]. Briefly, cells were seeded in a 96-well microplate (170,000 cells/mL). After one day of incubation, half of the cells were pretreated with ouabain (0.12 mM) and veratridine (0.012 mM) to evaluate cytotoxicity in the presence of CTXs when exposed to fish samples and CTX1B standard solution. After 20 h of incubation, cell mortality was assessed with MTT [3-(4,5-dimethylthiazol-2-yl)-2,5-diphenyltetrazolium] and DMSO (dimethyl sulfoxide) solutions. The absorbance was measured at 570 nm in a microplate spectrometer scanner (for further details, see Supplementary Materials).

### 5.7. Liquid Chromatography–Mass Spectrometry Analysis (LC-MS/MS and LC-HRMS)

Liquid chromatography–tandem mass spectrometry (LC-MS/MS) analysis was carried out in all groupers and extracts with cleaned up feed using the basic method described by Murata et al. [60] (further details are included in Supplementary Materials, Tables S3 and S4).

The presence of CTXs in samples was evaluated using Multiple Reaction Monitoring (MRM) mode for the following compounds: CTX1B; CTX2; CTX3; CTX4A; CTX3C; CTX3B; M-seco-CTX3C; 2,3-diOH-CTX3C; 51-OH-CTX3C; C-CTX1; 56-CH_2_-C-CTX1; 17-OH-C-CTX1; 17-OH-56-CH_2_-C-CTX1; and C-CTX3/4 (for details see Supplementary Materials Table S5). For confirmation of the signals detected in samples, these were compared with standard solutions and internal reference material by toxin retention time and MRM ion ratios according to EU Commission 2021/808 2021 [62] guidance document.

Quantification of the signals in the samples was accomplished by comparison with the CTX1B standard calibration curve for analogues of the C-CTX group, as no commercially pure C-CTX1 standard is available and there is a lack of an adequately quantified reference material with which to make an equivalency. A total of 8 levels of calibration were used (0.14 to 28 ng CTX1B/mL) with linear adjustment of R^2^ = 0.99 and a deviation slope of 12.5%. Therefore, the LOQs of the samples were 0.002 and 0.008 ng CTX1B/g of flesh and liver tissue, respectively. For CTX3C, 7 levels of calibration were used (0.28 to 28 ng CTX3C/mL) with R^2^ = 0.98 and a deviation slope of 12%, and LOQs 0.004 and 0.016 ng CTX3C/g Eq. of flesh and liver tissue, respectively.

LC-HRMS was reserved for confirmation of the signals detected by LC-MS/MS. Signals of CTXs were analyzed according to Tudó et al. [58]. Briefly, mass accuracy of de typical diagnostic ions for these molecules ([M + H]^+^, [M + NH_4_]^+^, [M + Na]^+^ as principal ions and [M + H − nH_2_O]^+^ as additional ions that support the detection of the above) was set as <5 ppm, and higher values for this parameter were taken into account when the following situations occurred: other accompanying adducts meet the mass accuracy or when the detected signal is weak but coincided with the retention time of a reference sample. Other confirmation points were restricted elements for molecular formula and adduct signals of CTXs (C: 55 to 70, H: 64 to 110, O: 11 to 25, N: 0 to 1, Na: 0 to 1), tolerance of ± 2.5% of retention time compared with reference compound, and the relative abundance (RA) between the main signals and their M+1 ions (approx. 0.7 for CTXs) was calculated and matched by taking into account a tolerance of 40% according to the following calculation as described by EU Commission 2021/808 2021: [RA (%) = [(M/M + 1 ion theorical) − (M/M + 1 ion measured)]/(M/M + 1 ion theorical)] [62]. Additionally, this last criterion does not necessary discard the signals, but it provides the qualification of suspected CTX if the other parameters are met, a situation that is normal when the signal is very low (further details are included in the Supplementary Materials, Tables S6 and S7).

### 5.8. Data and Statistical Analysis

Results are reported as mean values ± standard deviation. Data were adjusted to meet the requirements of normality of variance when the amount of data permitted. Differences were evaluated using the nonparametric Kruskal–Wallis test for multiple comparisons, the Mann–Whitney test for two independent samples, and Spearman’s correlation coefficient for correlations. In all analyses, differences were considered significant when *p* < 0.05. Statistical analysis was performed using SPSS software (Version 21.0, IBM Corp., Armonk, NY, USA). Linear and logarithmic regression analyses were conducted in GraphPad Prism 9.0 (GraphPad Software, San Diego, CA, USA).

In order to assess the relationship between CTX accumulation and toxicity in different tissues, both experimental groups (A and B) were integrated in the same figure (Figure 3). It should be noted that both groups differed in terms of the toxicity potential of the food supplied and, consequently, in the amount of toxin ingested over time. However, the total amount of CTX (ng Eq. CTX1B) was used as a common variable. In this way, data from both groups could be compared on a unified axis independent of exposure time, allowing identification of global trends in tissue accumulation.

## Figures and Tables

**Table 1:** Toxic burden and accumulation of CTXs in the flesh of the grouper during the experiment assessed by CBA.

Group (Toxic Food) ng Eq. CTX1B/g	Week	Fish (no.)	Total Quantity of CTX Ingested ^a^	Flesh			Liver		
				CTX-Like Toxicity ng Eq. CTX1B/g (n = 8) ^b^	Toxin Burden ^c^	CTXs Ingested Accumulated (%) ^d^	CTX-Like Toxicity ng Eq. CTX1B/g (n = 4)	Toxin Burden ^b^	Toxins Ingested Accumulated (%) ^c^
A 0.109 ± 0.003	4	1	52.69	<LOD	0	0	0.397 ± 0.032	4.85	9.22
		2	36.03	0.004 ± 0.002	1.02	2.85	0.782 ± 0.490	11.65	32.34
	10	3	107.77	0.012 ± 0.002	3.74	3.47	0.715 ± 0.150	10.72	9.95
		4	65.53	0.007 ± 0.003	1.95	2.97	1.085 ± 0.179	16.21	24.74
	18	5	218.48	0.049 ± 0.001	17.27	7.91	0.490 ± 0.325	13.48	6.17
		6	98.73	0.014 ± 0.006	2.63	2.66	0.779 ± 0.118	9.42	9.54
B 0.015 ± 0.001	6	7	10.78	<LOD	0	0	0.057 ± 0.008	0.87	8.09
		8	18.49	<LOD	0	0	0.032 ± 0.018	0.48	4.47
	12	9	29.73	<LOD	0	0	0.430 ± 0.311	8.90	29.96
		10	23.77	<LOD	0	0	0.120 ± 0.041	2.08	8.78

^a^ Calculated as the sum of ng eq. CTX1B ingested throughout the experiment from the toxicity of the food supplied. ^b^ Values are expressed as ng Eq. CTX1B/g of tissue ± SD (standard deviation) of the 4 flesh and 2 liver extracts prepared (2 CBA results per replicates per extract). ^c^ Calculated by multiplying CTX concentration by the weight of tissue stored per fish. ^d^ Calculated using the total amount of toxin ingested and tissue toxin burden.

**Table 2:** C-CTX analogues detected by LC-MS/MS in the different tissues from experimental fish from groups A and B expressed as ng Eq. CTX1B/g of tissue ± SD * (LC-MS assessment was conducted in the first replicate of extracts).

Group	Week	Fish (no.)	Flesh		Liver	
			C-CTX1 (ng)	17-OH-CTX1 (ng)	C-CTX1 (ng)	17-OH-CTX1 (ng)
A	4	1	-	-	0.082 ± 0.009	0.014 ± 0.0011
		2	-	-	0.071 ± 0.003	0.012 ± 0.0005
	10	3	-	-	0.167 ± 0.0003	0.030 ± 0.0006
		4	-	-	0.153 ± 0.006	0.028 ± 0.0008
	18	5	0.006 ± 0.0003	-	0.025 ± 0.002	-
		6	-	-	0.130 ± 0.007	0.018 ± 0.0014
B	6	7	-	-	0.020 ± 0.0003	-
		8	-	-	0.013 ± 0.0005	-
	12	9	-	-	0.024 ± 0.002	-
		10	-	-	0.015 ± 0.001	-

(*) Standard deviation; (-) <LOD.

**Table 3:** Tissue-specific assimilation efficiencies of C-CTXs normalized by daily intake rate. Comparison between *E. marginatus* and *L. rhomboides* dietary experiments.

Trial	Species	Tissue	Somatic Indexes (% ± SD)	Daily Intake of CTX Per g of Live Weight (pg CTX/g/d) ^a^	Uptake Rate Per g of Tissue Eq. (pg CTX/g/d) ^b^	Toxin Assimilation Rate Per Unit of Toxin Ingested, Corrected by Somatic Index (%) ^c^
Current experiment	*E. marginatus* (carnivore)	Muscle	20.33 ± 4.19	Group A = 1.4	0.29	4.18
		Liver	1.03 ± 0.19	Group B = 0.2	3.87	19.95
Bennet and Robertson [46]	*L. rhomboides* (omnivore)	Muscle	34.9 ± 6.78 ^d^	19 and 17	3.4	6.98 and 6.25
		Liver	1.00 ± 0.27 ^d^		63	3.71 and 3.32

^a^ Values expressed as pg CTX1B equivalents for current study and CTX3C for reference study [46]. ^b^ Time unit for uptake rate was converted from weeks to days to allow for comparison. ^c^ (uptake rate/daily intake) × corresponding somatic index. ^d^ Somatic indexes for experimental *L. rhomboides* were calculated from data available in the corresponding Supplementary Materials [46].

## Data Availability

The original contributions presented in this study are included in the article/Supplementary Materials. Further inquiries can be directed to the corresponding author.

## Supplementary Materials

The following supporting information can be downloaded at https://www.mdpi.com/article/10.3390/toxins18010003/s1, Figure S1: Results by CBA from experimental group B; Figure S2: Chromatograms for C-CTX1; Figure S3: Chromatograms for 17-OH-C-CTX1; Figure S4: Experimental design; Table S1: Toxicokinetic results; Table S2: LC-HRMS results; Table S3: Chromatographic conditions (LC-MS/MS); Table S4: Mass Spectrometry conditions (LC-MS/MS); Table S5: MS/MS conditions (MRM); Table S6: Chromatographic conditions (LC-HRMS); Table S7: Spectrometry assessment (LC-HRMS) [58,61,63].
