# Supplementary material for: C-CTX1 and 17-OH-C-CTX1 Accumulation in Muscle and Liver of Dusky Grouper (Epinephelus marginatus, Lowe 1834): A Unique Experimental Study Under Low-Level Exposure
Source: Toxins (Basel). 2025 Dec 19;18(1):3. doi: 10.3390/toxins18010003 (PMC12845688; doi:10.3390/toxins18010003)
Supplement: Supplementary file 1 [file toxins-18-00003-s001.zip › toxins-3992738-supplementary.pdf]

C-CTX accumulation in muscle and liver of dusky grouper: a unique experimental study under low-level exposure

Index

Results by CBA from experimental group B 2

Toxicokinetic results 3

Results by LC-MS/MS 4

Results by LC-HRMS 6

Materials and Methods 7

Experimental design 7

Neuro-2a Cell-Based Assay for CTX Determination 8

Liquid chromatographic-mass spectrometry analysis (LC-MS/MS) 9

Liquid chromatographic-high resolution mass spectrometry analysis (LC-HRMS) 13

Supporting information

Results by CBA from experimental group B

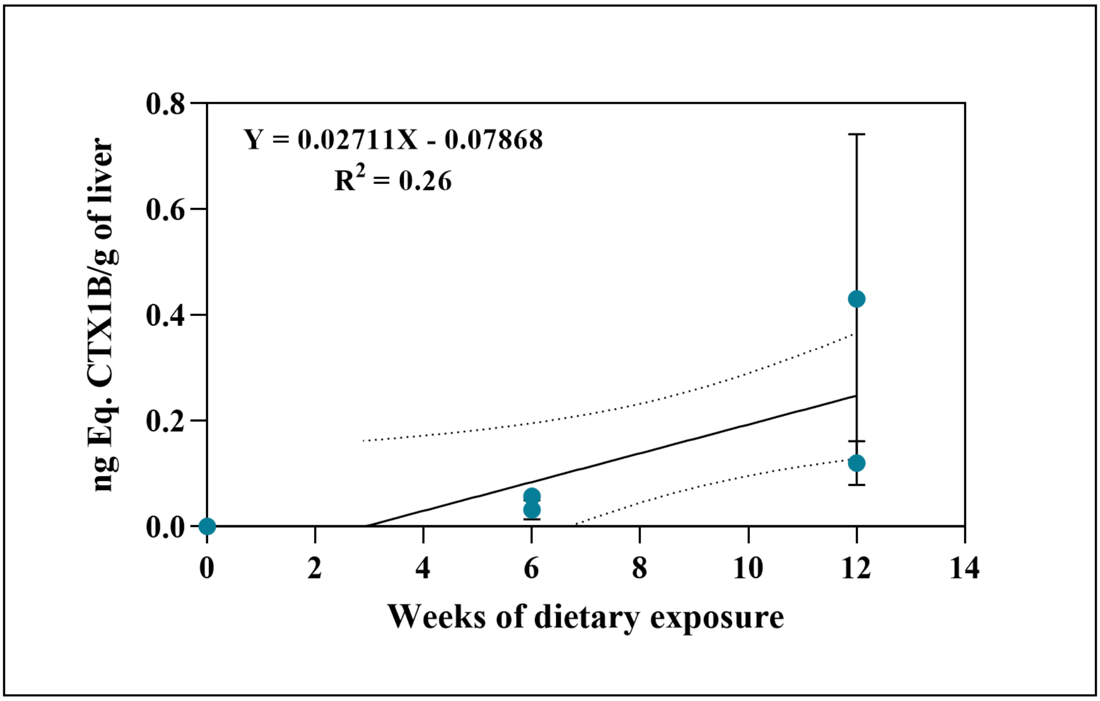

Figure S1. CTX-like toxicity (ng Eq. CTX1B/g of liver) values in liver per fish by sampling week (6 and 12 weeks) from group B, assessed by CBA. The dashed lines indicate the 95% confidence interval of the linear fit.

Toxicokinetic results

Table S1. Model fitting parameters and uptake kinetics describing toxin bioaccumulation in muscle and liver tissues of groups A and B.

Group	Tissue	Statistical parameters	Bioaccumulation equation<br>Logarithmic: $Y = A \times (1 - e^{-kt})$<br>Linear: $Y = mx + b$	Uptake rate (pg/g/weeks)	t_f (weeks) ($t = - \ln (1 - f)/k$)
A	Liver	Exponential plateau	$Y = 0.7599 \times (1 - e^{-0.5844t})$	-	$t_{25\%} = 0.5$
		R^2	0.27		$t_{50\%} = 1.2$
		95% CI	A = 0.0.625 to 0.9086		$t_{90\%} = 3.9$
			k = 0.1840 to (not converged)		
		p - value	0.69		
	Flesh	Linear regression	Y = 0.00201X – 0.00735	2.0	-
		R^2	0.53		
		95% CI	m = 0.00144 to 0.00259		
		p - value	< 0.0001*		
B	Liver	Linear regression	Y = 0.02711X – 0.07868	27.1	-
		R^2	0.26		
		95% CI	m = 0.00419 to 0.05002		
		p - value	0.023*		

* p-value ≤ 0.05 is significant.

Results by LC-MS/MS

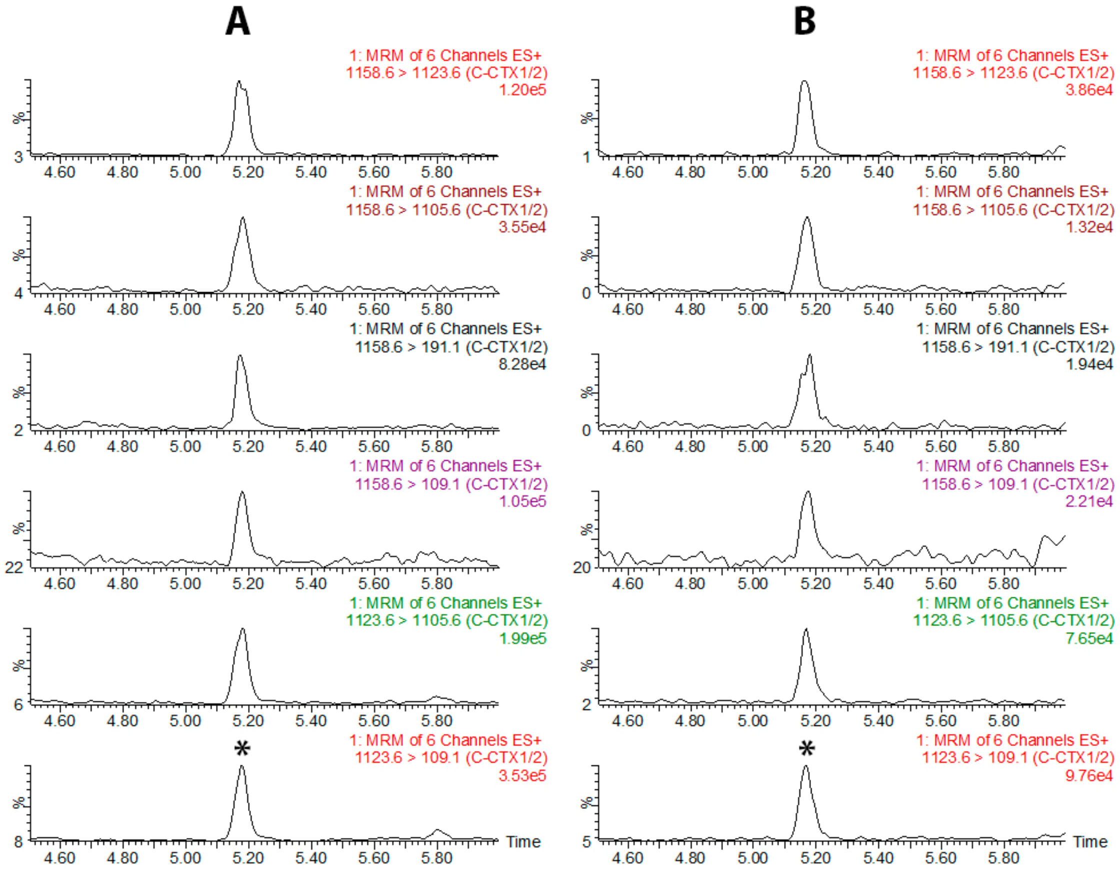

Figure S2. Multiple Reaction Monitoring (MRM) chromatograms for C-CTX1. **A**: Liver from *Seriola* sp. internal reference material used for retention time and ion ratio confirmation. **B:** Liver from experimental fish no. 3. (*) Signal considered for quantification.

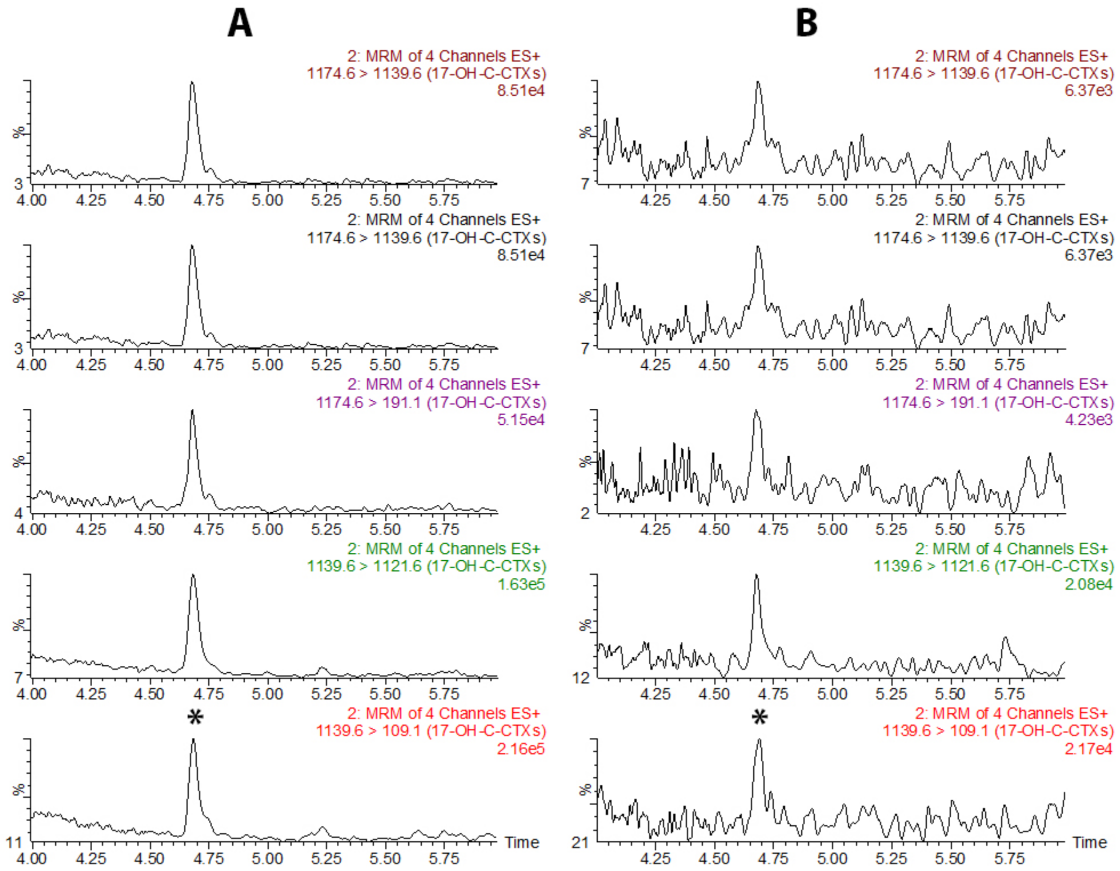

Figure S3. Multiple Reaction Monitoring (MRM) chromatograms for 17-OH-C-CTX1. A: Liver from *Seriola* sp. internal reference material used for retention time and ion ratio confirmation. B: Liver from experimental fish no. 3. (*) Signal considered for quantification.

Results by LC-HRMS

Table S2. Parameters measured on the C-CTX analogues detected by LC-HRMS in *Seriola* sp. reference material and liver samples from experimental group A (first replicate of extracts).

	C-CTX1			17-OH-C-CTX1				
	$[M + NH_4]^+$ (m/z 1158.6571)			$[M + NH_4]^+$ (m/z 1174.6520)			$[M + H - H_2O]^+$ (m/z 1139.6149)	
Sample	tR[1] (min)	Mass accuracy (ppm)	RA[2] (M+1)	tR[1] (min)	Mass accurac y (ppm)	RA[2] (M+1)	Mass accuracy (ppm)	RA[2] (M+1)
Seriola sp. Reference material	4.06	2.843	-3.0%	2.51	4.980	-7.3%	7.189	N.D.
Liver Exp. Fish no. 3	4.07	3.559	-46.3%	2.49	4.282	-58.3%	-	-
Liver Exp. Fish no. 4	4.06	3.154	-43.3%	2.43	8.819	N.D.	-	-
Liver Exp. Fish no. 6	4.08	4.880	-43.0%	2.45	1.727	N.D.	-	-

[1] tR: retention time; [2] relative abundance of monoisotopic ion, criteria stablished at ± 40% of tolerance; N.D.: no date.

Materials and Methods

Experimental design

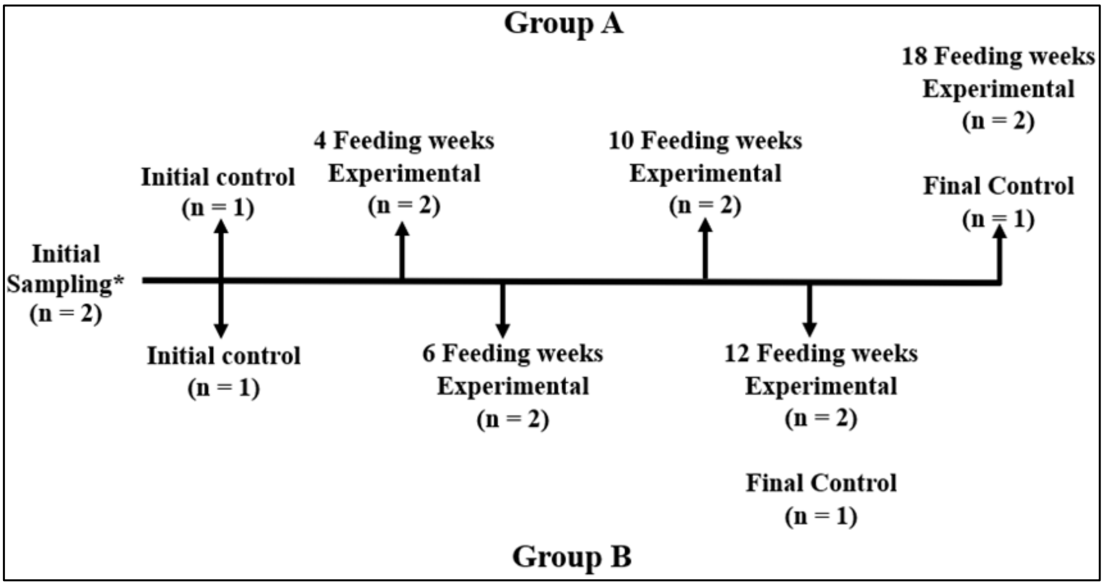

Figure S4. Scheme of each sample and temporal duration of the dietary exposure period to CTXs by feeding group. In the upper timeline, the sampling from group A is represented, and in the lower part of the line, group B is represented. (*) Corresponds to the initial sampling prior to the conditioning period.

Neuro-2a Cell-Based Assay for CTX Determination

The assay chosen to determine the toxicity of the feed and fish tissue samples of the groupers was the Neuro-2a-MTT assay. For the assay and cell maintenance, we followed the protocol by Caillaud et al. [61]. The cell line used was Neuroblastoma 2a (N2a, CCL131, from ATCC, LGC Standards SLU, Barcelona, Spain). To assess the CTX-like toxicity, CTX1B standard solution was obtained from R.J. Lewis (University of Queensland, Brisbane, Queensland, Australia) and was used to compare its response with the fish samples.

Briefly, the cells were seeded in a 96-well plate at a concentration of 170,000 cells/mL; half of the microplate was pretreated with ouabain (0.12 mM) and veratridine (0.012 mM) to evaluate cytotoxicity in the presence of CTX. Then, the cells were exposed to the fish samples and CTX1B solution standard in decreasing concentrations to obtain a dose–response curve. The calibration standard curve of CTX1B was used to calculate the limit of detection and quantification (LOD/LOQ) when there was 20% of inhibition of cell viability (IC_{20}) compared with the maximum concentration of the extract when no unspecific toxicity was observed. CTX-like toxicity measure was given in CTX1B Eq. and was calculated based on the IC_{50} value of the sample, when it is achieved, and the IC_{50} CTX1B standard curve. A cell mortality inferior to 20% in a sample is considered a negative result for CTX-like activity [1]. Samples (flesh, liver, and food extracts) were analyzed in duplicate on different days when the volume of the extract permitted. The CTX1B standard dose–response curve was obtained each day the samples were analyzed (IC_{50} = 0.620 ± 0.137 pg CTX1B/mL). Cell viability was assessed using MTT [3-(4,5-dimethylthiazol-2-yl)-2,5-diphenyltetrazolium] and DMSO solutions.

Once the assay was ended, the absorbance was measured at 570 nm using a microplate spectrometer scanner (Agilent® BioTek Synergy LX multi-mode reader) and processed in Microsoft Excel 2021 (Microsoft Corporation) and GraphPad Prism 9 (GraphPad, California, USA) software.

Liquid chromatographic-mass spectrometry analysis (LC-MS/MS)

For this assessment, a Xevo™ TQ-XS coupled to an Acquity™ UPLC I-Plus-Class (both from Waters® Corporation, Milford, MA, USA) with N_2 supplied by a generator NM20Z (Peak® scientific, Renfrewshire, Scotland, UK) were used. The analytical chromatography and mass spectrometry assessment conditions were carried out according to the basic method described by Murray et al. [63] (detailed in Tables S3 and S4) using an Acquity™ UPLC BEH Phenyl column (100 mm * 2.1 mm, 1.7 µm particle size, from Waters® Corporation, Milford, MA, USA) at 50 °C and sample injection volume of 2 µL at 8 °C.

Table S3. Chromatographic conditions in Acquity™ UPLC I-Plus-Class (LC-MS/MS).

**Time** (min)	**Mobile phase A** H_2O + 0.2% of NH_4OH (25%)	**Mobile phase B** Acetonitrile-H_2O (95:5) + 0.2% of NH_4OH (25%)	**Flow** (mL/min)
0	95%	5%	0.55
1	95%	5%	0.55
3.5	50%	50%	0.55
7.5	25%	75%	0.55
8	5%	95%	0.55
9	5%	95%	0.55
9.2	95%	5%	0.55
10	95%	5%	0.55

Table S4. Mass spectrometry conditions in Xevo™ TQ-XS (LC-MS/MS).

Ionization mode	Capillary voltage	Source temperature	Desolvation temperature	Gas flow desolvation	Cone gas flow	Nebulizer gas flow
ESI* (+)	3.5 kV	150 °C	600 °C	N_2 at 1000 L/h	300 L/h	7 bar

(*) ESI: electrospray ionization. Control and acquisition software: MassLynx™ V4.2 and TargetLynx™ XS (Waters® Corporation)

Table S5. MS/MS conditions. Multiple Reaction Monitoring (MRM) transitions implemented in standards and reference material in the present study by LC-MS/MS.

CTX analogue (Acronym) Chemical formula	Transition	Parent (m/z)	Daughter (m/z)	Cone (V)	Collision Energy (eV)
CTX1B $C_{60}H_{86}O_{19}$	$[M+NH_4]^+$ > 95*	1128.6	95	20	70
	$[M+NH_4]^+$ > 109	1128.6	109	50	70
	$[M+Na]^+$ > $[M+Na]^+$	1133.6	1133.6	20	20
52-epi-54-deoxy-CTX1B (CTX2) $C_{60}H_{86}O_{18}$	$[M+NH_4]^+$ > 125*	1112.6	125	50	55
	$[M+NH_4]^+$ > 155	1112.6	155	20	45
	$[M+NH_4]^+$ > $[M+H-2H_2O]^+$	1112.6	1077.6	20	20
54-deoxy-CTX1B (CTX3) $C_{60}H_{86}O_{18}$	$[M+NH_4]^+$ > 125*	1112.6	125	40	55
	$[M+NH_4]^+$ > 155	1112.6	155	20	45
	$[M+NH_4]^+$ > $[M+H-2H_2O]^+$	1112.6	1077.6	20	20
52-epi-CTX4B (CTX4A) and CTX4B $C_{60}H_{84}O_{16}$	$[M+H]^+$ > 125.1*	1061.6	125.1	70	55
	$[M+H]^+$ > 155	1061.6	1043.6	50	55
	$[M+Na]^+$ > $[M+Na]^+$	1083.6	1083.6	15	20
CTX3C and 49-epi-CTX3C (CTX3B) $C_{57}H_{82}O_{16}$	$[M+H]^+$ > 125.1*	1023.6	125	30	55
	$[M+H]^+$ > 155.1	1023.6	155.1	30	50
	$[M+Na]^+$ > $[M+Na]^+$	1045.6	1045.6	20	20
M-seco-CTX3C $C_{57}H_{84}O_{17}$	$[M+H-H_2O]^+$ > 125.1*	1023.6	125.1	60	55
	$[M+H-H_2O]^+$ > 155.1	1023.6	155.1	70	45
2,3-dihydroxy-CTX3C (2,3-diOH-CTX3C) $C_{57}H_{84}O_{18}$	$[M+H]^+$ > 125.1*	1074.6	125.1	50	55
	$[M+H]^+$ > 155.1	1074.6	155.1	60	45
	$[M+H]^+$ > $[M+H-H_2O]^+$	1057.6	1039.6	20	20
51-hydroxy-CTX3C (51-OH-CTX3C) $C_{57}H_{82}O_{17}$	$[M+H]^+$ > 95*	1039.6	95	20	60
	$[M+H]^+$ > 141.1	1039.6	141.1	40	90
	$[M+H]^+$ > 171.1	1039.6	171.1	10	45
	$[M+H]^+$ > $[M+H-H_2O]^+$	1039.6	1021.6	20	20
C-CTX1 $C_{62}H_{92}O_{19}$	$[M+H-H_2O]^+$ > 109.1*	1123.6	109.1	10	70
	$[M+H-H_2O]^+$ > $[M+H-2H_2O]^+$	1123.6	1005.6	20	20
	$[M+NH_4]^+$ > $[M+H-H_2O]^+$*	1158.6	1123.6	60	20
	$[M+NH_4]^+$ > $[M+H-2H_2O]^+$	1158.6	1005.6	30	25
	$[M+NH_4]^+$ > 109.1	1158.6	109.1	10	70
	$[M+NH_4]^+$ > 191.1	1158.6	191.1	30	45
56-methoxy-C-CTX1 (56-CH_2-C-CTX1) $C_{63}H_{94}O_{19}$	$[M+H-CH_2-H_2O]^+$ > 109.1*	1123.6	109.1	10	70
	$[M+H-CH_2-H_2O]^+$ > 191.1	1123.6	191.1	30	45
	$[M+H-CH_2-H_2O]^+$ > $[M+H-CH_2-2H_2O]^+$	1123.6	1105.6	20	20
	$[M+NH_4]^+$ > $[M+H-H_2O]^+$	1172.6	1137.6	20	20
17-hydroxy-C-CTX1 (17-OH-C-CTX1) $C_{62}H_{92}O_{20}$	$[M+H-H_2O]^+$ > 109.1*	1139.6	109.1	70	70
	$[M+H-H_2O]^+$ > $[M+H-2H_2O]^+$	1139.6	1121.6	90	20
	$[M+NH_4]^+$ > $[M+H-H_2O]^+$	1174.6	1139.6	50	20
	$[M+NH_4]^+$ > 191.1	1174.6	191.1	60	45
17-hydroxy-56-methoxy-C-CTX1 (17-OH-56-CH_2-C-CTX1) $C_{63}H_{94}O_{20}$	$[M+H-CH_2-H_2O]^+$ > 109.1*	1139.6	109.1	70	70
	$[M+H-CH_2-H_2O]^+$ > $[M+H-CH_2-2H_2O]^+$	1139.6	1121.6	90	20
C-CTX3 and C-CTX4 $C_{62}H_{94}O_{19}$	$[M+H]^+$ > 108.9*	1143.6	108.9	30	70
	$[M+H]^+$ > $[M+H-H_2O]^+$	1143.6	1125.6	60	20
	$[M+NH_4]^+$ > $[M+H]^+$	1160.7	1143.6	20	15

Gas collision: Ar at 0.15 mL/min; (*) quantification transition.

Liquid chromatographic-high resolution mass spectrometry analysis (LC-HRMS)

For this analysis, a Orbitrap-Exactive HCD (Thermo Fisher Scientific Bremen, Germany) mass spectrometer, coupled to a Surveyor MS plus Pump and an Accela Open AS auto-sampler kept isothermal (15 °C) (from Thermo Fisher Scientific, San José, CA, USA), was used under conditions proposed by Tudó et al. [58] and detailed in Tables S4 and S5. The chromatography was carried out on a Hypersil Gold C18 column (100 mm * 2.1 mm, 1.9 μm particle size, from Thermo Fisher Scientific, Bremen, Germany) for 5 μL of injection sample. Acquisition data were processed with Xcalibur 3.0.63 software (Thermo Fisher Scientific, Bremen, Germany).

Table S6. Chromatographic conditions in Surveyor MS plus Pump (LC-HRMS).

**Time** (min)	**Mobile phase A** H_2O + 0.2 mM of ammonium formate + 0.1% formic acid	**Mobile phase B** Acetonitrile- H_2O (95:5) + 0.2 mM of ammonium formate + 0.1% formic acid	**Flow** (mL/min)
0	50%	50%	0.25
1	50%	50%	0.25
9	10%	90%	0.25
9.1	0%	100%	0.25
12	0%	100%	0.25
25	50%	50%	0.25

Table S7. Spectrometry assessment conditions in Orbitrap-Exactive HCD.

Ionization mode	Resolutio n	Acquisition mode	Mass range	Ion spray voltage	Capillary temp.	Heater temp.
ESI* (+)	50,000	Full scan	400 to 1500 m/z	4 kV	275 °C	300 °C

Sheath gas	Auxiliary gas	Capillary voltage	Tube lens	Max. Inject time	Automatic gain control
N_2 at 35 psi	N_2 at 10 psi	47.5 V	186 V	250 msec	"Balanced " (1x106)

(*) ESI: electrospray ionization. Acquisition data and processing software: Xcalibur 3.0.63 (Thermo Fisher Scientific)
